# Successful outcome of disseminated *Candida tropicalis* osteomyelitis on remission induction for childhood Philadelphia chromosome–positive acute lymphoblastic leukaemia-case report

**DOI:** 10.1186/s13052-020-00953-x

**Published:** 2021-02-11

**Authors:** Lichun Xie, Qingling Long, Guichi Zhou, Sixi Liu, Fei-Qiu Wen

**Affiliations:** 1 The First Affiliated Hospital, Jinan University, Guangzhou, Guangdong China; 2 Department of Hematology/Oncology, Shenzhen Children’s Hospital, No. 7019 Yitian Rd, Shenzhen, Guangdong China

**Keywords:** *Candida tropicalis*, Case report, Dasatinib, Immunocompromised, Osteomyelitis, Philadelphia chromosome–positive acute lymphoblastic leukaemia

## Abstract

**Background:**

Invasive fungal infection (IFI) is one of the most challenging complications in children undergoing acute lymphoblastic leukaemia (ALL) treatment, but acute fungal osteomyelitis (OM) is rarely encountered.

**Case presentation:**

Here, we describe a case of *Candida tropicalis* osteomyelitis in a 10-year-old patient with Philadelphia chromosome (Ph)-positive ALL. He was on remission induction therapy at the time of neutropenia, and an abscess developed in his right arm. The blood and bone cultures were positive for *C. tropicalis*. Antibiotics and antifungals were administered. Magnetic resonance imaging of the arm revealed an intraosseous abscess, suggestive of OM. Surgical irrigation and debridement of the bone were performed immediately. The patient was effectively treated with antifungal therapy and ALL treatment. He has fully recovered into complete clinical remission but with visible sequelae on magnetic resonance imaging (MRI). He took oral posaconazole for consolidation until disappearance of the lesion shadows on MRI and received subsequent cycles of chemotherapy in parallel.

**Conclusions:**

In the successful management of Ph-positive ALL, dasatinib, a second-generation Abl-tyrosine kinase inhibitor, is crucial. The recommended treatment for *Candida* osteomyelitis in Ph-positive ALL patients is a fungicidal agent combined with surgery and modification chemotherapy with dasatinib. The use of combined modalities of treatment seems to be crucial in the successful management of Ph-positive ALL.

## Background

Acute lymphoblastic leukaemia (ALL) is the most common childhood malignancy [1]. Children with ALL are immunocompromised patients who are at high risk of infection associated with high morbidity and mortality rates [2]. Invasive fungal infection (IFI) is one of the most challenging complications in children with ALL treatment [3], but acute fungal osteomyelitis (OM) is rarely encountered. In immunocompromised children, the most common mechanism of these complications includes fungal osteomyelitis due to continuous infiltration or haematogenous spread [4]. *Candida* species are weak pathogens that are common commensal organisms inhabiting the skin and mucous membranes of most individuals. However, the rate of incidence of invasive *Candida* infection is increasing among newborns in the ICU and in immunosuppressive therapy, the augmented use of intravenous catheters and haematological malignancy and neutropenia in children [5]. Successfully managing fungal osteomyelitis and chemotherapy for ALL is quite difficult. Here, we report a case of childhood Philadelphia chromosome (Ph)-positive acute lymphoblastic leukaemia, complicated by disseminated *Candida tropicalis* osteomyelitis of the right humerus during remission induction that was successfully managed by timely and appropriate administration without compromising anti-leukaemia therapy.

## Case presentation

A 10-year-old boy was confirmed to have Philadelphia chromosome (Ph)-positive ALL. According to the Chinese Children’s Cancer Group study ALL-2015 (CCCG-ALL-2015), he was provisionally assigned as intermediate-risk based on his presenting clinical features, aged 10 years, and immunophenotype B- cell and Philadelphia chromosome–positive ALL. According to CCCG-ALL-2015, the patient will receive MRD directed, risk-stratified treatment, modified from the St Jude Children’s Research Hospital Total XV and XVI studies and the Shanghai Children’s Medical Center ALL-2005 trial. For MRD ≥ 1% at the end of remission induction, provisional intermediate-risk patient has to be escalated to high risk. Allogeneic transplant was recommended. He received upfront window therapy with dexamethasone for 4 days followed by remission induction with prednisone acetate, vincristine, daunorubicin hydrochloride (DNR), and pegaspargase from days 5 to 28.

**Fig. 1:**
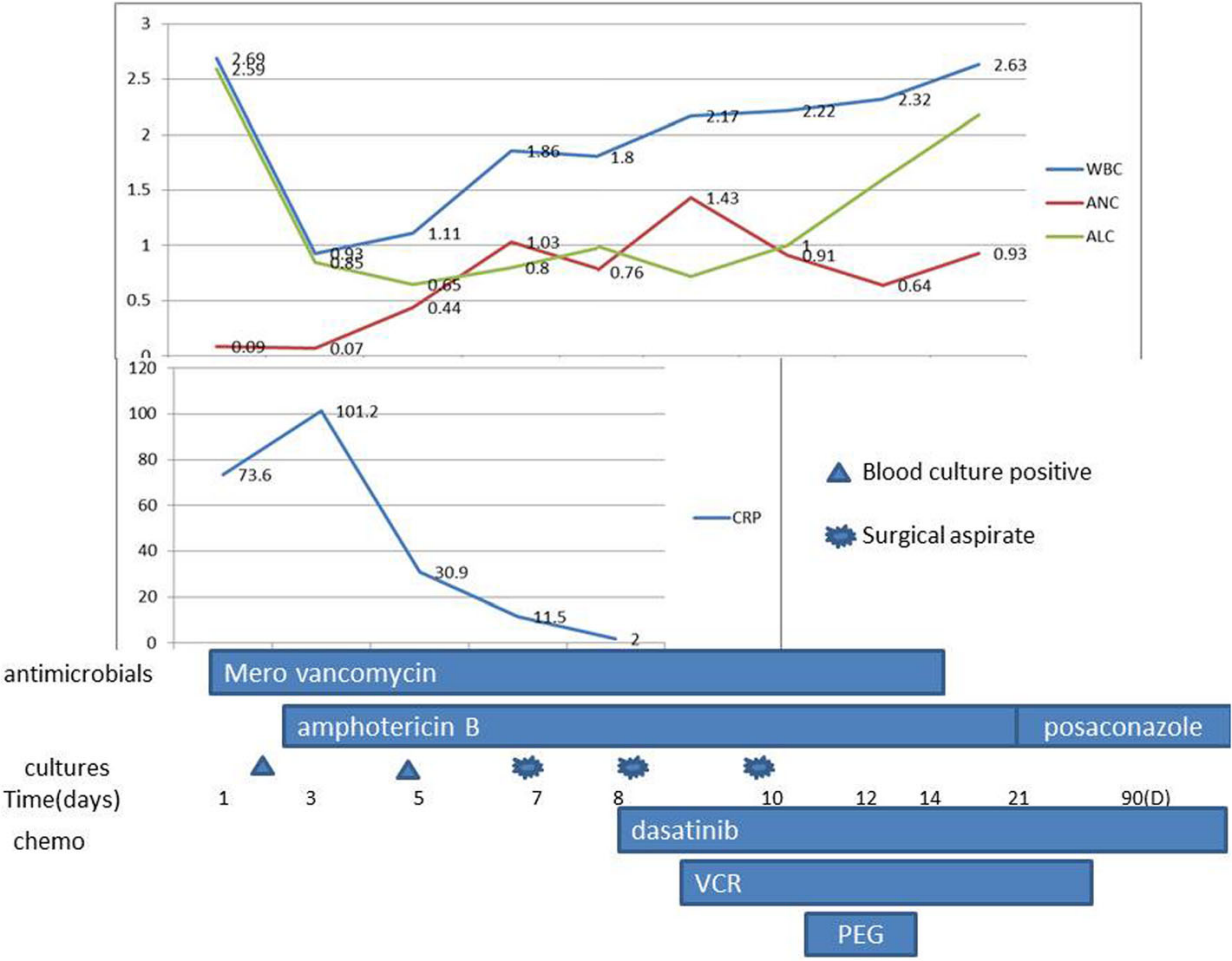
The results of the laboratory test, antimicrobial treatment and chemotherapy

**Fig. 2:**
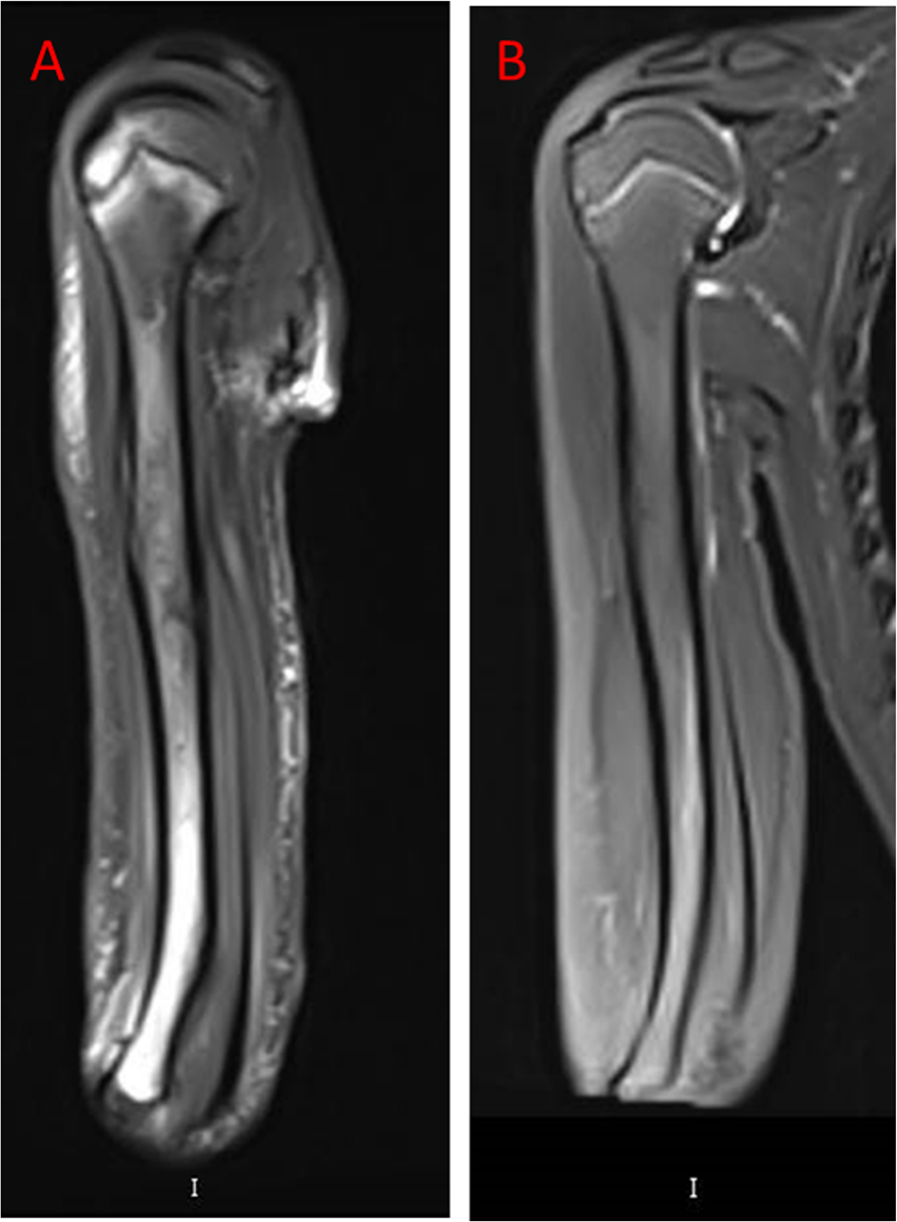
The MRI results of the right arm at first (**a**). At the follow-up MRI, the previous abnormal signals narrowed significantly (**b**)

**Fig. 3:**
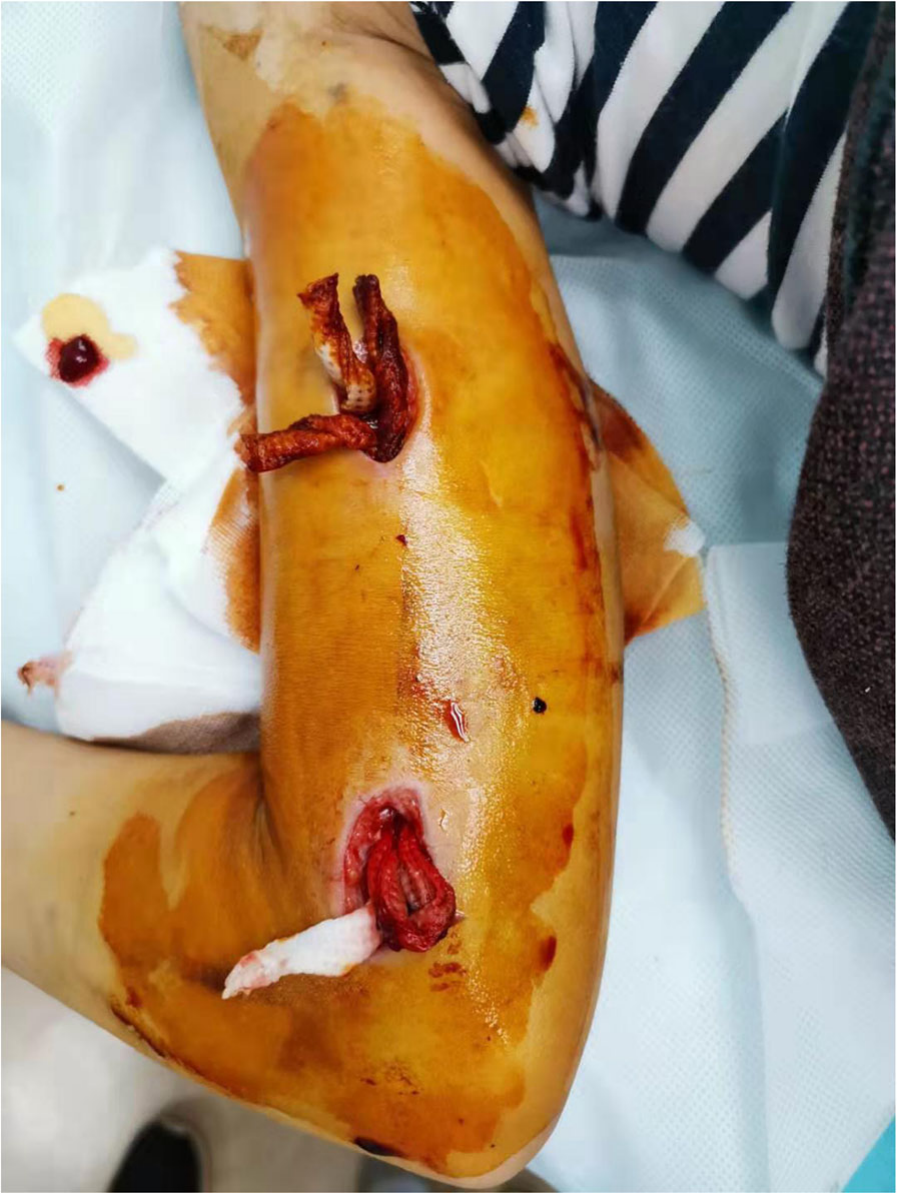
Surgical irrigation and debridement of the bone

He began to receive dasatinib (80 mg/m^2^ per day) as soon as the diagnosis was made on day 5 of remission induction. On day 18 of remission induction, he was hospitalized for cough, fever, and pain, tenderness and swelling over the right elbow. Laboratory tests were notable for neutropenia and elevated inflammatory markers (Fig. 1). A computed tomography (CT) chest scan showed the patchy shadows in the anterior lobe of left upper lung and the middle lobe of right lung. The right upper limb ultrasound revealed swelling of soft tissue without previous trauma. Empiric treatment with broad-spectrum antibiotics was initiated to cover both gram-positive and gram-negative bacteria. The lesion progressed to an abscess in the upper right arm. Twenty-two hours later, blood culture showed fungus positivity and pus. Empirical amphotericin B therapy was initiated. There are two reasons we chose amphotericin B. First, *Candida* infection is the most common fungal infection. Second, vincristine is one of the drugs used in remission induction chemotherapy. Voriconazole may increase the toxic side effects of vincristine, so voriconazole was not our first option [6]. The blood culture results suggested *C. tropicalis* 2 days later. Minimal inhibitory concentrations indicated susceptibility to amphotericin B (minimal inhibitory concentration < =0.5 μg/mL), fluconazole (1) and voriconazole (0.125) and flucytosine (<=0.4). He subsequently developed pain and swelling over the right upper arm and forearm. Five days after admission, he was afebrile, but pain, oedema, and limited function of the right elbow were progressive. Repeated ultrasound revealed discrete periosteal oedema adjacent to the humerus 1 week after admission. Magnetic resonance imaging (MRI) of the right humerus showed an intraosseous abscess and probable osteomyelitis (Fig. 2). Surgical irrigation and debridement of the bone were performed immediately (Fig. 3). Culture of bone aspirates obtained during surgery showed that *C. tropicalis* was sensitive to amphotericin B, which is the same result as the blood culture. A small study of the bone fragments collected during surgery revealed fungal spores. All chemotherapy, except prednisone was continued, was withdrawn for 1 week. Debridement was repeated on the third postoperative day, resulting in considerable improvement of clinical and laboratory findings. His condition improved. One week later, when he was nontoxic, haemodynamically stable, with no signs of haematopoietic toxicity and inflammatory markers within the normal range, induction chemotherapy was continued with some modification with prednisolone and dasatinib, and treatment was carried out for osteomyelitis with amphotericin B, meropenem and vancomycin in parallel. Successful control of osteomyelitis was achieved by serial surgical debridement of the lesion, antibiotics, amphotericin B and HBO therapy. Treatment with amphotericin B was administered for a total of 3 weeks; during this time, he regained the ability to move his arm and transitioned to oral posaconazole for consolidation, which he is currently receiving on an ongoing basis. Vincristine (D19 and D26) and L-asparaginase (D26) were administered until the fever was controlled and the neutrophil count (ANC) exceeded 500/mm^3^. After completion of remission induction therapy, he achieved complete remission (CR). Then, we removed the remission induction treatment consisting of cyclophosphamide (CTX), cytarabine (Ara-c), and mercaptopurine (6-MP) from days 29 to 35. The next chemotherapy regimen was continued with dasatinib, weekly vincristine and daily mercaptopurine for 3 weeks. Subsequent cycles of chemotherapy were reinitiated as per the protocol. At follow-up 5 months later, the patient continued to feel good.

## Discussion and conclusions

*Candida* are found on the skin and in the respiratory tract, vagina and stools. According to a multicentre study in Australia [7], *Candida* species accounted for the majority of infections associated with neutropenia in immunocompromised patients with ALL, of which *Candida albicans* and *Candida parapsilosis* were most frequently detected. According to another retrospective study, the proportion of *Candida* is increasing in children with ALL [8]. To our knowledge, there are few reports of *C. tropicalis* osteomyelitis in a child with ALL. Predisposing factors present in our case were underlying leukaemia, steroids, neutropenia, and immunosuppressive therapy, which is consistent with the literature report [9].

Proving a diagnosis is the first key step in the successful management of IFI. Isolating and identifying the causative fungus on culture is important for epidemiological and research purposes. The culture of specimens from surgery is more sufficient than blood to determine the pathogen and helps to establish the diagnosis as quickly as possible.

A combination of early diagnosis, surgical debridement and antifungal therapy seems to be crucial. The timely administration of empirical amphotericin B on suspicion of fungal infection in our case along with debridement of necrotic tissue may be responsible for a successful outcome.

In the successful management of Ph-positive ALL, in the pre-TKI era, matched donor allogeneic stem cell transplantation was beneficial [10]. Sequential addition of imatinib (a tyrosine kinase inhibitor) to the chemotherapy regimen in Ph-positive ALL dramatically improved the outcomes for children with Ph-positive ALL [11]. TKIs administered in the early phases of therapy can dramatically improve the outcome of childhood Ph-positive ALL. According to the study, there was no significant difference in 5-year disease-free survival (DFS) for patients who received imatinib alone versus patients who received imatinib followed by HSCT [12]. Dasatinib is a crucial second-generation Abl–tyrosine kinase inhibitor. According to our randomized clinical trial, dasatinib at a dosage of 80 mg/m^2^ per day yielded superior results in the treatment of Ph-positive ALL compared with imatinib mesylate at a dosage of 300 mg/m2 per day [13]. Anti-infection combined with surgical incision and drainage and dasatinib administration together led to the successful treatment of osteomyelitis while maintaining a remission state of Ph-positive ALL.

The importance of judicious concomitant administration of antifungal and anti-leukaemia treatment during induction therapy in ALL is highlighted. Moreover, we emphasize that the most efficient diagnostic and treatment strategy for patients with fungal osteomyelitis with ALL is the combination of medical and surgical management. When there is significant marrow toxicity and infection in a patient with Ph-positive ALL, concurrent myelosuppressive agents such as DNR, Ara-C, CTX, MTX, and 6-MP should be stopped instead of dasatinib. It can be an effective treatment option for patients who need both infection control and ALL treatment.

## Data Availability

The microarray data will be available in the NCBI database.

## Abbreviations

ALL: Acute lymphoblastic leukaemia
Ara-c: Cytarabine
CCCG-ALL-2015: Chinese Children’s Cancer Group study ALL-2015
CT: Computed tomography
CR: Complete remission
CTX: Cyclophosphamide
DNR: Daunorubicin hydrochloride
IFI: Invasive fungal infection
OM: Osteomyelitis
Ph: Philadelphia chromosome
ANC: Neutrophil granulocyte
MRI: Magnetic resonance imaging
6-MP: Mercaptopurine
